# Development of a Method for the Quantification of Clotrimazole and Itraconazole and Study of Their Stability in a New Microemulsion for the Treatment of Sporotrichosis

**DOI:** 10.3390/molecules24122333

**Published:** 2019-06-25

**Authors:** Patricia Garcia Ferreira, Carolina Guimarães de Souza Lima, Letícia Lorena Noronha, Marcela Cristina de Moraes, Fernando de Carvalho da Silva, Alessandra Lifsitch Viçosa, Débora Omena Futuro, Vitor Francisco Ferreira

**Affiliations:** 1 Departamento de Tecnologia Farmacêutica, Faculdade de Farmácia, Universidade Federal Fluminense, Niterói-RJ 24241-000, Brazil; patricia.pharma@yahoo.com.br (P.G.F.); leticianoronha95@gmail.com (L.L.N.); dfuturo@id.uff.br (D.O.F.); 2 Departamento de Química Orgânica, Instituto de Química, Universidade Federal Fluminense, Niterói-RJ 24210-141, Brazil; carolgslima@gmail.com (C.G.d.S.L.); mcmoraes@id.uff.br (M.C.d.M.); gqofernando@vm.uff.br (F.d.C.d.S.); 3 Fundação Oswaldo Cruz (FIOCRUZ), Farmanguinhos-Manguinhos, Avenida Sinzenando Nabuco 100, Rio de Janeiro-RJ 21045-900, Brazil; alessandra.vicosa@far.fiocruz.br

**Keywords:** pre-development process, clotrimazole, itraconazole, stability, method validation, sporotrichosis

## Abstract

Sporotrichosis occurs worldwide and is caused by the fungus *Sporothrix brasiliensis*. This agent has a high zoonotic potential and is transmitted mainly by bites and scratches from infected felines. A new association between the drugs clotrimazole and itraconazole is shown to be effective against *S. brasiliensis* yeasts. This association was formulated as a microemulsion containing benzyl alcohol as oil, Tween^®^ 60 and propylene glycol as surfactant and cosurfactant, respectively, and water. Initially, the compatibility between clotrimazole and itraconazole was studied using differential scanning calorimetry (DSC), thermogravimetric analysis (TG), Fourier transform infrared spectroscopy (FTIR), and X-ray powder diffraction (PXRD). Additionally, a simple and efficient analytical HPLC method was developed to simultaneously determine the concentration of clotrimazole and itraconazole in the novel microemulsion. The developed method proved to be efficient, robust, and reproducible for both components of the microemulsion. We also performed an accelerated stability study of this formulation, and the developed analytical method was applied to monitor the content of active ingredients. Interestingly, these investigations led to the detection of a known clotrimazole degradation product whose structure was confirmed using NMR and HRMS, as well as a possible interaction between itraconazole and benzyl alcohol.

## 1. Introduction

Sporotrichosis is a subcutaneous infectious disease with subacute to chronic evolution and with a worldwide distribution. The etiologic agent of sporotrichosis is *Sporothrix schenckii*, which is a thermo-dimorphic fungus that lives saprophytically in nature and is pathogenic to humans and animals [1,2]. The occurrence of sporotrichosis in animals, especially cats, as well as its transmission to humans has been reported in several countries [3]. In this context, the Brazilian state of Rio de Janeiro is an epidemic area for this disease and the first one associated with zoonotic transmission related to sick felines by *Sporothrix brasiliensis*, the most virulent species from the *S. schenckii* complex [4].

**Figure 1:**
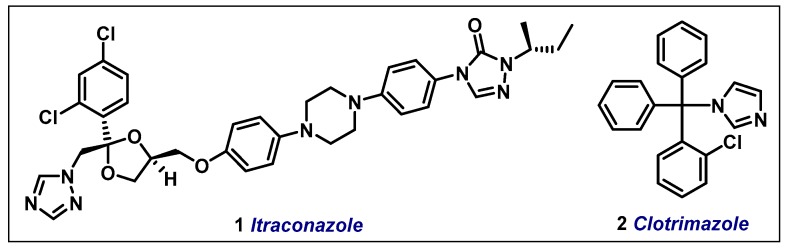
Chemical structures of clotrimazole and itraconazole.

The treatment of both feline and human sporotrichosis is based on the use of itraconazole 1, which contains the 1,2,4-triazole scaffold in its structure and inhibits the synthesis of sterol, a vital component of the fungus cell membrane [5,6]. Clotrimazole 2, on the other hand, is an imidazole derivative with antifungal activity that is only indicated for topical use due to its toxicity (Figure 1). Similarly to itraconazole, clotrimazole is a synthetic antifungal and its mechanism of action involves the inhibition of sterol biosynthesis [7]. In this sense, Gagini et al. [8] reported the effectiveness of the combination of itraconazole with clotrimazole against *S. brasiliensis* yeasts (the infective form) from feline and human sporotrichosis isolates, suggesting that clotrimazole by itself or in combination with itraconazole is potentially a new option for the treatment of sporotrichosis.

Accordingly, the development of new pharmaceutical technologies for the use of clotrimazole and itraconazole associations is highly desirable in order to increase their efficiency in therapy, decrease adverse effects and provide, especially for felines, alternative treatments. Moreover, the use of a combination antifungal therapy is a promising approach to avoid resistance [9]. Allied to all the mentioned features, the association of known drugs is highly advantageous for the pharmaceutical industry to find innovations for the market, since they can reformulate their products in a more economically advantageous way when compared to the development of new drugs. In addition, the association of drugs already in use in the pharmaceutical market may increase their efficiency with known safety and effectiveness, reintroducing forgotten and/or discarded ones.

Considering the development of new formulations, microemulsions (MEs) have attracted great interest as potential drug delivery systems, mainly due to their unique physicochemical properties such as drug solubilization and enhanced absorption properties [10,11]. MEs are a thermodynamically stable, isotropic, transparent liquid system consisting of two immiscible liquids (usually water and oil) stabilized by a film of surfactant compounds, suitably combined with a cosurfactant [12,13]. The presence of the surfactant helps to reduce the interfacial tension, making it possible to join the oil and aqueous phases [14,15]. MEs have been proposed as an innovative formulation approach to improve solubility and efficacy and reduce of the toxicity of various drugs. Therefore, when the known hydrophobicity of clotrimazole and itraconazole are taken into account, such systems could be particularly advantageous for their delivery.

In light of the aforementioned concepts, this paper reports the initial research phase for the pre-development of a clotrimazole–itraconazole formulation, the first step towards a new antifungal combination. In this sense, the development and characterization of this new pharmaceutical formulation requires the evaluation of parameters such as drug release and stability. Therefore, as a further extension of our work in the field, we have developed a simple, sensitive, and specific HPLC method for the simultaneous quantification of clotrimazole and itraconazole in microemulsion. Although many researchers have investigated clotrimazole and itraconazole singly or in combination with other compounds, to the best of our knowledge, no HPLC method has been developed for the simultaneous determination of both drugs simultaneously, especially in microemulsion systems [16,17]. Finally, we performed an accelerated stability study of this formulation and the developed analytical method was applied to monitor the content of active ingredients. Interestingly, these investigations led to the detection of a known clotrimazole degradation product whose structure was confirmed using NMR and HRMS, as well as a possible interaction between itraconazole and benzyl alcohol.

## 2. Results and Discussion

### 2.1. Study of the Compatibility between Clotrimazole and Itraconazole

We initiated our studies by analyzing the physicochemical properties of both active ingredients as well as their compatibility using different techniques such as thermal analyses (differential scanning calorimetry (DSC) and thermogravimetric/derivative thermogravimetry (TG)/DTG) analysis), powder X-ray diffraction (XRD) and FTIR.

Initially, we proceeded to characterize the active ingredients and their combination using thermal analyses, which offer the ability to quickly screen for potential drug–drug incompatibilities. Such interactions can be of a physical or chemical nature and may affect the stability and bioavailability of the final product, compromising the therapeutic efficacy and safety [18].

**Figure 2:**
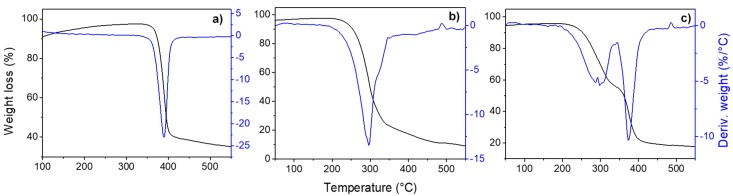
Thermogravimetric (TG) and derivative thermogravimetry (DTG) curves for (**a**) clotrimazole, (**b**) itraconazole and (**c**) the binary mixture of clotrimazole and itraconazole (1:1).

The TG and DTG curves of clotrimazole (Figure 2a) showed that it is thermally stable up to 340 °C, when its thermal decomposition starts; the highest rate of weight loss occurs at 388.6 °C, as showed in the DTG curve, and is finished at 421.1 °C, where a loss of 60% of the total weight is observed. As for itraconazole, its thermal decomposition starts at 200 °C and is finished at 348.4 °C, with a maximum rate at 295.3 °C and a total weight loss of 87%. The TG profile of the binary mixture of clotrimazole and itraconazole (1:1 ratio) showed two decomposition steps, indicating that the compounds undergo thermal degradation independently, although a small shift in the initial temperature of decomposition was observed, as expected.

Next, the DSC technique was employed to further analyze the occurrence of events related to possible interactions between the drugs [19]. It is noteworthy that although such analyses are conducted upon heating the sample to high temperatures, which is not consistent with the process of drug production nor its administration to patients, they afford important information regarding the physical properties of the sample [18].

**Figure 3:**
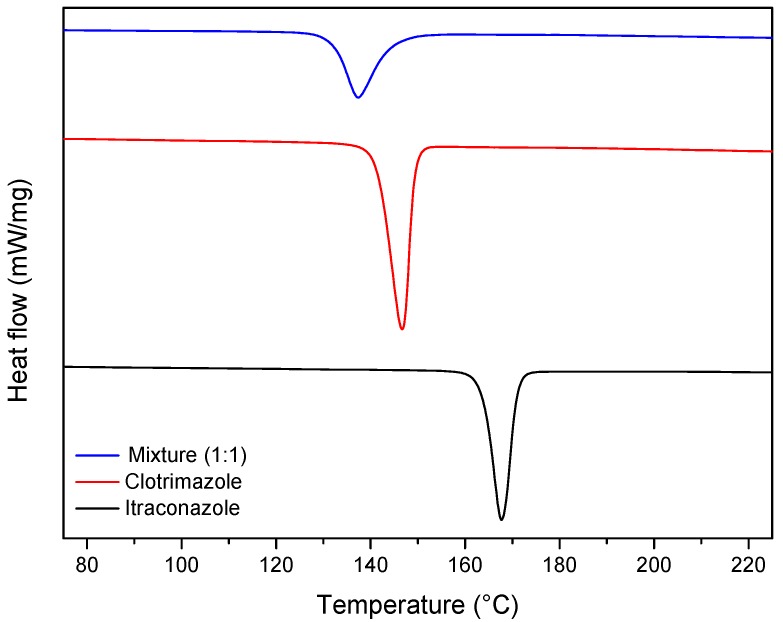
Differential scanning calorimetry (DSC) profile of itraconazole, clotrimazole, and the clotrimazole/itraconazole binary mixture (1:1).

The DSC curves of the drugs showed endothermic peaks attributed to the melting of the drugs between 158.5 and 175.0 °C (ΔH = 31.5 J g^−1^) for itraconazole and 136.8 and 153.1 °C (ΔH = 41.6 J g^−1^) for clotrimazole. On the other hand, a single endothermic event was observed in the DSC curve of the binary mixture, starting at 127.7 and finishing at 137.1 (ΔH = −25.35 J g^−1^), which suggests a strong interaction between clotrimazole and itraconazole (Figure 3).

**Figure 4:**
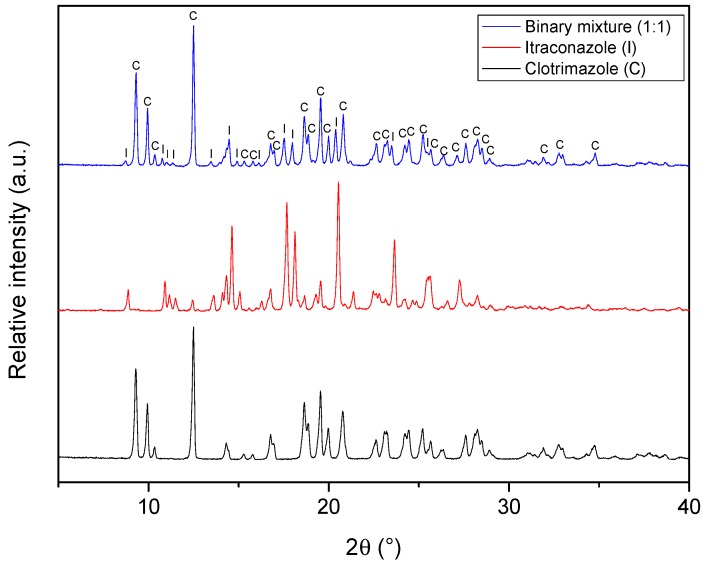
X-ray diffractograms of clotrimazole, itraconazole, and the binary mixture (1:1).

In order to further explore the possibility of interactions between the active ingredients, powder X-ray diffraction (PXRD) analyses were conducted. Interestingly, the diffractogram of the binary mixture (Figure 4) contained virtually all the peaks of clotrimazole and itraconazole, with no marked displacement of the peaks being observed. Furthermore, it is important to highlight that it was not possible to notice the appearance of any new peaks, which means that if there is any interaction between the drugs, it probably is not strong enough to take place in the solid state. The same observations were made in the FTIR spectra of the binary mixture, which showed the characteristic bands observed for the isolated active ingredients (For more details, see the Supplementary Materials).

With the characterization of the active ingredients and the binary mixture in hand, we proceeded to develop an HPLC method for their quantification in a newly developed microemulsion for the treatment of sporotrichosis.

### 2.2. Determination of the Concentration of Clotrimazole and Itraconazole in Microemulsions Using HPLC Analyses

Considering the unique properties presented by microemulsions, in the present work, benzyl alcohol was used as an oil phase, Tween^®^ 60 as a surfactant, and propylene glycol as a cosolvent in the presence of water. These components were chosen on the basis in their previously reported applications in other pharmaceutical forms available on the international market.

**Figure 5:**
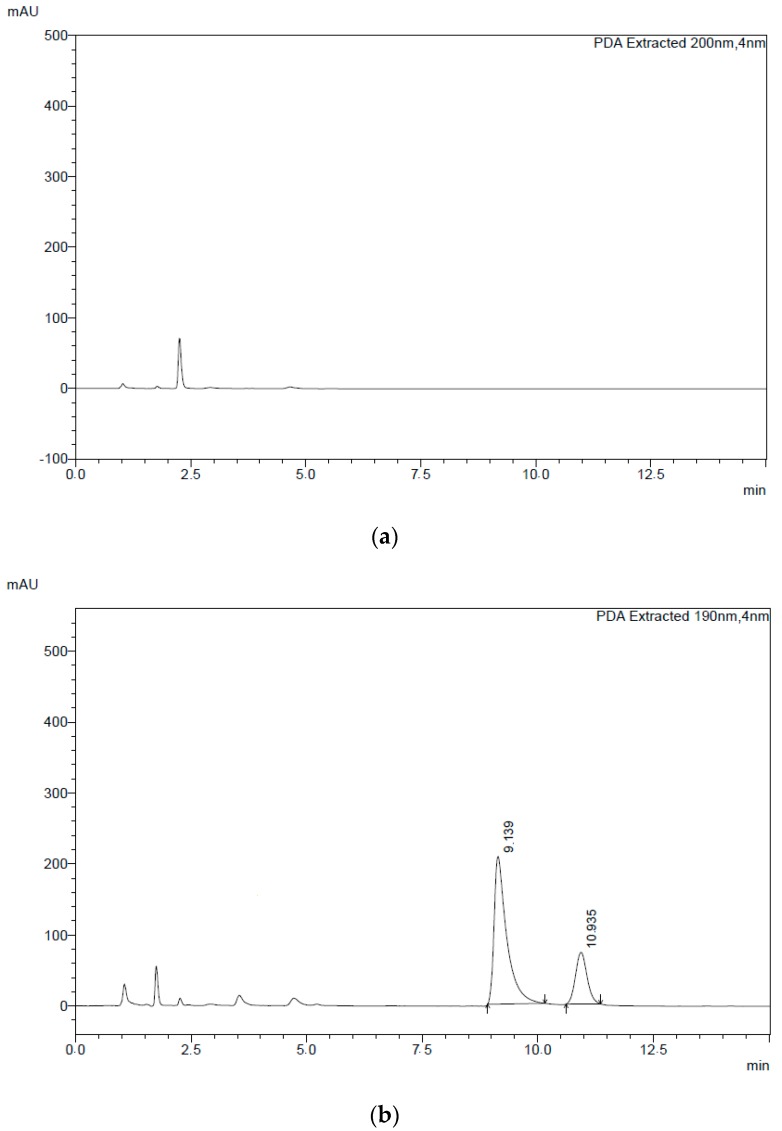
Chromatograms of the (**a**) mobile phase and (**b**) standard solution containing a binary mixture of itraconazole and clotrimazole.

In this context, HPLC-DAD (diode array detector) was selected as an analytical tool for the simultaneous quantification of clotrimazole and itraconazole in the developed microemulsion through a rapid, simple, and isocratic method [20]. In our study, the best separation condition was achieved using a C18 analytical column with a mobile phase composed of acetonitrile and a phosphate buffered saline 0.05 M (pH 8.0 with ammonium hydroxide 1 M) in the ratio (*v*/*v*) 60:40, respectively, with a 1 mL min^−1^ flow rate and UV detection at 190 nm. A typical chromatogram is presented in Figure 5, with a retention time of 9.1 min being observed for clotrimazole and 10.9 min for itraconazole.

To evaluate the linearity of the method, calibration standards of clotrimazole (5–200 µg mL^−1^) and itraconazole (5–160 µg mL^−1^) were analyzed. A linear relationship was established for the injected concentration ranges versus the peak area for both analytes, with determination coefficients greater than 0.9988 (see the calibration curves in the Supplementary Materials). The calibration curve parameters are reported in Table 1, with the linearity parameters of the method shown in Table 2.

**Figure 6:**
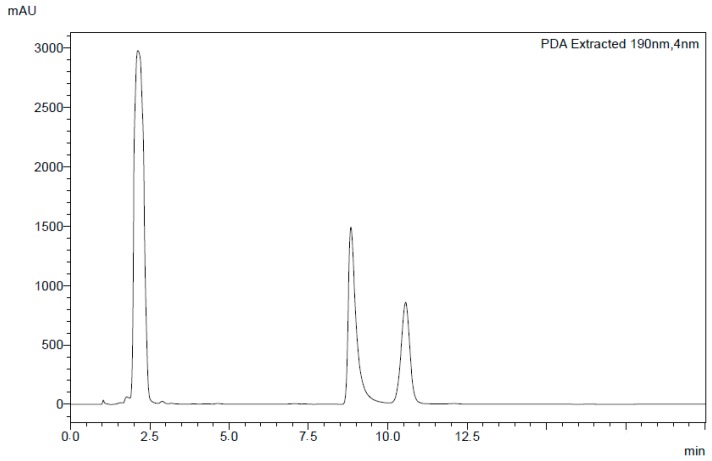
Chromatogram obtained from the injection of the microemulsion using the developed HPLC method.

The method’s selectivity was confirmed by the absence of interferences at the retention times of itraconazole and clotrimazole in the microemulsion prepared without the drugs (Figure 6). The purity of the compounds was checked using PDA (photodiode array) detection. The within-assay precision (repeatability) was carried out by performing six consecutive analyses of standard solution at three different concentrations for each drug on the same day. The samples were also analyzed on different days to evaluate the between-assay precision (intermediate precision). The obtained values were evaluated through the dispersion of the results by calculating the standard deviation of the measurement series. The intra- and inter-day precision relative standard deviation (RSD %) was between 1.18 and 0.8 for clotrimazole and 1.48 and 0.84 for itraconazole. The recovery of the drugs was in the range of 93.8–100.9% with RSDs below 2.35% for clotrimazole and in the range of 100.5–104.3% with RSDs below 2.40% for itraconazole. The results are given in Table 3.

No changes were observed in the drug concentrations of the stock solutions under storage conditions. Indeed, further analyses showed that the percent recovery of clotrimazole and itraconazole were, respectively, 97.3% ± 3.15 and 91.3% ± 2.71 at room temperature (25 °C) and 94.2 ± 0.34 and 88.7 ± 1.63 under refrigeration (−5 °C, Table 4). Moreover, the drugs were stable for at least 30 days under storage conditions, with RSDs below 8%.

**Figure 7:**
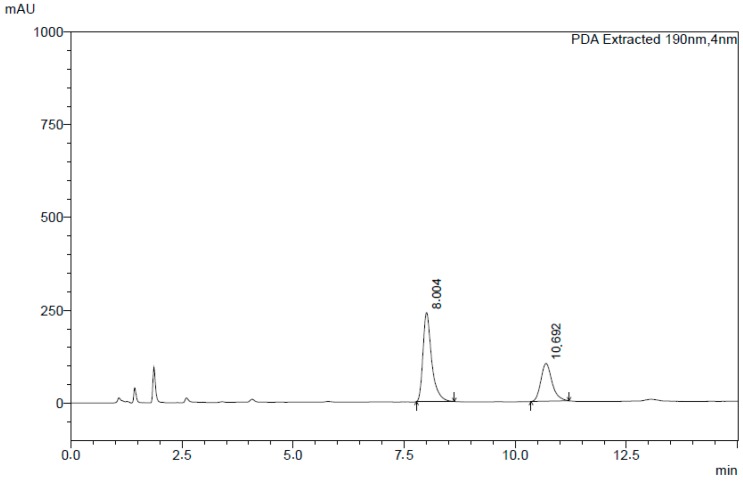
Chromatogram of clotrimazole and itraconazole obtained in the robustness studies.

In order to evaluate the robustness of the chromatographic method, assays were carried out by changing both the column brand and ratio of the mobile phase for acetonitrile 70:30 (*v*/*v*) and a phosphate buffered saline 0.05 M (pH 8.0 with ammonium hydroxide 1 M). The alteration of the column brand and the mobile phase did not promote any significant variations in the retention time of clotrimazole and itraconazole peaks; a good resolution was observed with retention times of 8 min for clotrimazole and 10.7 min for itraconazole (Figure 7).

### 2.3. Study of the Stability of a Novel Microemulsion Containing Clotrimazole and Itraconazole

Subsequently, the developed method was used in the determination of clotrimazole and itraconazole in the newly developed microemulsion with the purpose of quantifying the drugs in the formulation, as well as in the accelerated stability study. Based on the assumption that possible interactions and incompatibilities may arise from the contact between the drugs over time, they were left to stand for three months, both under refrigeration and heating conditions, and further analyzed.

**Figure 8:**
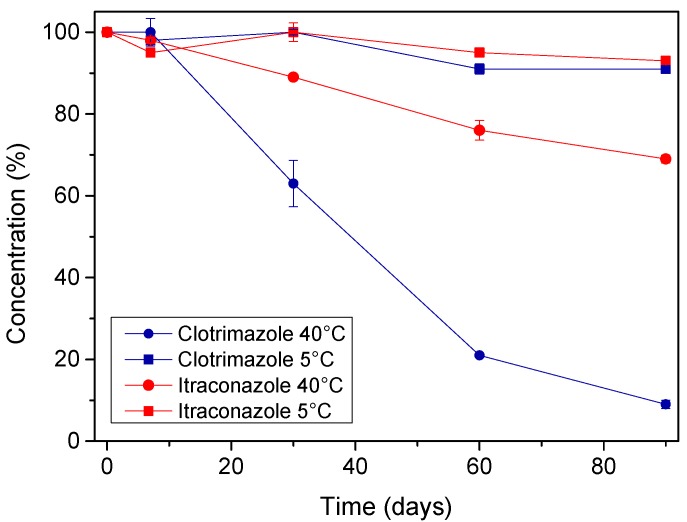
Graph showing the concentration of clotrimazole and itraconazole over time in different conditions. All data is represented as mean ± SD (n = 3).

**Figure 9:**
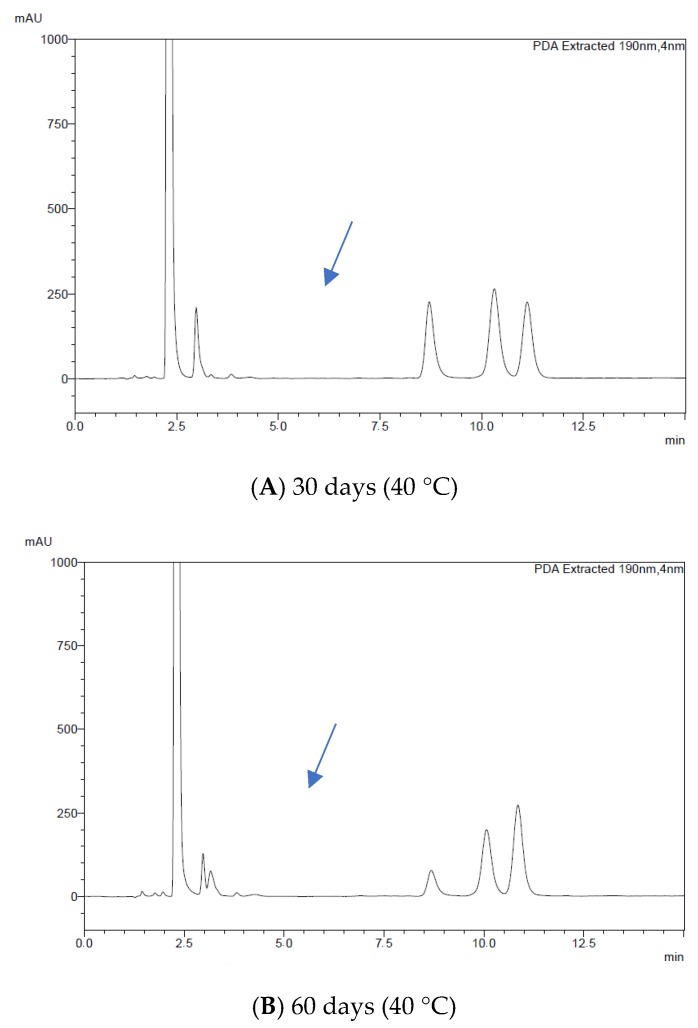

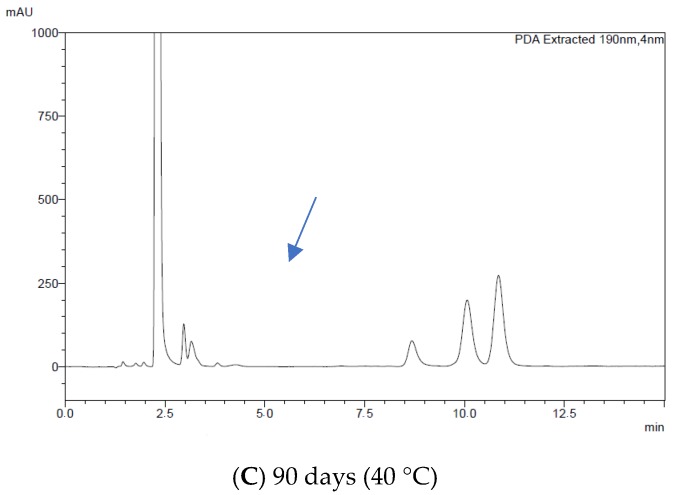
HPLC chromatograms for the samples in the stability study after (**A**) 30 days, (**B**) 60 days, and (**C**) 90 days.

The initial drug content of the microemulsion was taken as 100%, and the drug content over time was plotted (Figure 8), with all data being represented as mean ± SD (n = 3). For the samples stored at 5 °C, no significant changes were observed for both drugs when compared to the first day. Furthermore, it is noteworthy that there was no evident interaction between clotrimazole and itraconazole at this temperature, since the peaks of both drugs were detected independently without the appearance of any additional peaks. On the other hand, when the samples that were stored at 40 °C were analyzed, it was possible to notice a significant decrease in the concentration of the drugs over time, especially for clotrimazole. Additionally, a new peak could also be observed in the chromatogram of such samples (Figure 9).

**Figure 10:**
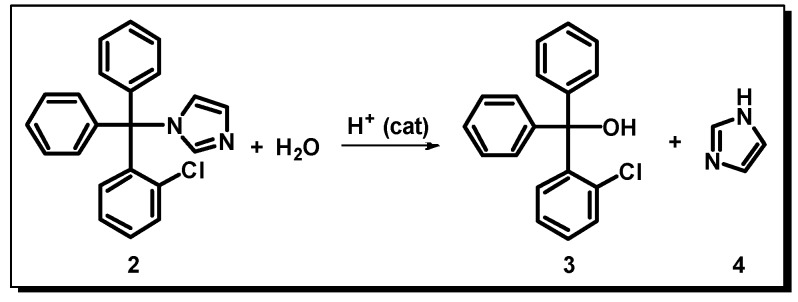
Reaction scheme showing the degradation of clotrimazole in acid medium.

In order to investigate the formation of this compound, which might be a result of the interaction between clotrimazole and itraconazole, we conducted further studies. Initially, we sought to investigate which degradation products could be formed from the degradation of both drugs and found that the degradation of clotrimazole is well-reported under acidic conditions, giving product **3** (Figure 10).

With these concepts in mind, we conducted the synthesis of compound **3** from clotrimazole by heating it at 80 °C in the presence of acetonitrile and concentrated hydrochloric acid for 2 h; the product identity was confirmed using NMR and HRMS by comparing the obtained data with previous reports (for details, see the Supplementary Materials) [21]. Next, we conducted the forced degradation of a mixture of itraconazole and clotrimazole by heating both at 50 °C for 24 h in a solution of acetonitrile, water, and benzyl alcohol-mimicking the microemulsion composition—and isolated the formed product using column chromatography.

With both compounds in hand, we analyzed product **3** and the degradation product by HPLC using the developed method, and the comparison of the retention times of both compounds proved that, indeed, product **3** is formed from the degradation of clotrimazole under acidic conditions. Furthermore, the retention time was also a match for the product previously detected in the stability studies conducted at 40 °C, which proves that under specific conditions, clotrimazole may undergo degradation in the presence of traces of acid, forming **3**. However, the formation of **3** was not observed in the stability studies conducted at 5 °C, which shows the viability of this novel microemulsion and encourages carrying out further studies for its development.

**Figure 11:**
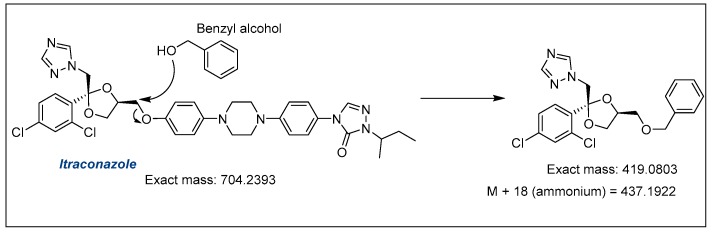
Scheme showing the reaction between itraconazole and benzyl alcohol.

The content of itraconazole (% *w*/*w*) in the microemulsions stored in climatic chambers also underwent a slight decrease, which was less significant when compared to clotrimazole. In order to exclude the possibility of interaction between the drugs, the decrease in itraconazole content was also investigated. However, unlike clotrimazole, degradation studies of itraconazole are not found in the literature. Thus, an aliquot was collected directly from the chromatographic system at the same retention time as the degradation product formed during the stability study; at a low intensity, it was possible to observe a product with a mass-to-charge ratio (*m*/*z*) of 437.1931. Considering that the itraconazole concentration change was lower than for clotrimazole, we hypothesized that an interaction of itraconazole with some other excipient of the microemulsion may be taking place. In that sense, we propose that the degradation product may be formed by the nucleophilic addition of benzyl alcohol to the methylene group linking the phenolic aromatic part with the 1,3-dioxolane ring (Figure 11); indeed, the mass-to-charge ratio was a match for the proposed product. It is worth mentioning that although we have observed a good match in a mass-to-charge ratio of 437.1931, further studies are necessary to confirm whether the proposed structure is indeed the correct one, such as the isolation and complete spectroscopic characterization of this compound, which was not possible at the scale we were working.

## 3. Experimental Methods

### 3.1. Materials for Analytical Method Development

Clotrimazole and itraconazole (as a mixture of stereoisomers) standards were purchased from Merck, São Paulo, SP, Brazil. Microemulsions were prepared using Tween^®^ 60, propylene glycol, and benzyl alcohol, all purchased from Merck, São Paulo, SP, Brazil. HPLC-grade acetonitrile was acquired from J.T. Baker Inc., Phillipsburg, NJ, USA. Clotrimazole (Jintan Zhongxing Pharmaceutical Chemical Co., Ltd., Mainland, China) and itraconazole (Metrochem API, Telangana, India) were donated by Valdequimica Produtos Quimicos Ltd., São Paulo, Brazil. All solutions were prepared with ultra-pure Milli-Q water obtained from a Milli-Q Water Millipore purification system (Burlington, MA, USA).

### 3.2. Compatibility Study of Clotrimazole and Itraconazole

#### 3.2.1. Preparation of Clotrimazole/Itraconazole Binary Mixtures

The binary mixtures were prepared and homogenized by taking clotrimazole and itraconazole in a 1:1 proportion (*w*:*w*). These mixtures were further used for X-ray powder diffraction, Fourier transform infrared spectroscopy (FTIR), and thermal analyses.

#### 3.2.2. X-ray Powder Diffraction (PXRD)

PXRD patterns were collected on a Bruker D8 Venture diffractometer system (Bruker, Billerica, MA, USA) operating at 1.5406 Å, 40 kV voltage, and a current of 40 mA using a Cu Kα radiation source. The samples were contained in a flat poly(methyl methacrylate) sample holder and the data acquisition was done in a range of 5 to 70° (2θ) at 0.019°/0.1 s step size over a total period of 10 min.

#### 3.2.3. Fourier Transform Infrared Spectroscopy (FTIR)

The FTIR spectra of the solid samples were obtained using a Varian FT-IR 660 equipment (Varian Inc., Walnut Creek, CA, USA). A hydraulic press was used to prepare pellets for analysis. The KBr pellets contained 3 mg of a sample and 100 mg of KBr. Spectra were collected with a resolution of 4 cm^−1^ on the spectral domain of 3800–600 cm^−1^.

#### 3.2.4. Thermal Analyses

DSC data were collected on a Shimadzu Differential Scanning Calorimeter DSC-60A (Shimadzu, Quioto, Japan). Approximately 4 mg samples were placed in aluminum pans, and the temperature program was set to increase from 30 to 250 °C with a heating rate of 10 °C min^−1^ under nitrogen flow (50 mL min^−1^).

Thermogravimetric (TG) analyses were performed using a Netzsch STA 409 PC/PG (Netzsch, Selb, Germany) under a nitrogen atmosphere with a flow rate of 60 mL min^−1^ at a heating rate of 10 °C min^−1^ over the range of 30 to 300 °C and using 6 mg of sample in an aluminum cell.

### 3.3. Instruments and Chromatographic Conditions

Chromatographic experiments were performed on a Shimadzu SPD-M20A system (Shimadzu, Quioto, Japan). The chromatographic separations were performed using a 150 mm × 4.6 mm i.d. (5 µm particle size) Fortis C18 column in isocratic elution mode with acetonitrile and phosphate buffered saline 0.05 M pH 8.0 adjusted with ammonium hydroxide 1 M (60:40, *v*/*v*) at a flow rate of 1.0 mL min^−1^. The detection wavelength was set at 190 nm, and the injection volume was 20 μL.

### 3.4. Standard Stock Solutions and Calibration Standards

Standard stock solutions of clotrimazole and itraconazole were freshly prepared by dissolving the drugs in methanol (0.2 mg mL^−1^) Calibration standards in the concentration range of 5, 10, 20, 40, 80, 160, and 200 µg mL^−1^ were prepared in the appropriate volumetric flasks by diluting the stock solution in the mobile phase. An aliquot (20 μL) of the solution was then directly injected into the HPLC.

### 3.5. Sample Preparation

An amount of microemulsion was accurately weighted to contain 25 mg clotrimazole and itraconazole in a 50 mL centrifuge tube and heated for 5 min in a water bath at 50 °C. The sample was then removed from the bath, shaken until cooled to room temperature, and placed in an ice-methanol bath. Next, the sample was centrifuged for 5 min and extracted with chloroform (5 mL). Finally, the solvent was removed under a stream of gaseous nitrogen, and the residue was diluted in the mobile phase.

### 3.6. Method Validation Protocol

The proposed method was validated under the optimized conditions regarding its linearity range, selectivity, sensitivity, precision, accuracy and stability of the assay according to the regulatory guidelines requirements (FDA).

#### 3.6.1. Linearity Range

The linearity range was evaluated by measuring the chromatographic peak area responses of the drugs at seven concentration levels and in triplicate. Analytical curves were constructed by plotting the peak area against the concentration of itraconazole and clotrimazole (Figure 2 and Figure 3), which gives the regression equation. The results are presented in Table 1.

#### 3.6.2. Selectivity

To ensure the selectivity of the proposed method, drug-free microemulsions were prepared and analyzed in the described chromatographic conditions.

#### 3.6.3. Sensitivity

The sensitivity was determined by means of the limit of detection (LOD) and limit of quantification (LOQ). One of the ways to calculate the LOD (Equation (1)) and LOQ (Equation (2)) is based on the standard deviation (σ) of the y-intercept from the regression of the calibration standard. The results are given in Table 1.

(1) $$LOD=\frac{3,3.\sigma }{s}$$

*LOD* (*σ*—standard deviation; *s*—slope of the calibration standard).

(2) $$LOQ=\frac{10.\sigma }{s}$$

*LOQ* (*σ*—standard deviation; *s*—slope of the calibration standard).

#### 3.6.4. Precision and Accuracy

The accuracy and precision of the method were estimated by quintuplicate quality control (QC) samples prepared using the mobile phase: 7 μg mL^−1^ (low QC), 15 μg mL^−1^ (medium QC), and 120 μg mL^−1^ (high QC) for clotrimazole and 7 μg mL^−1^ (low QC), 70 μg mL^−1^ (medium QC), and 150 μg mL^−1^ (high QC) for itraconazole. Accuracy was established through back-calculation and expressed as the percent difference between the found and the nominal concentration for each compound, and the precision was calculated as the coefficient of variation (CV) of the replicate measurements. Calibration standards and QC samples were analyzed in three different batches in order to determine the intra and inter-batch variability.

#### 3.6.5. Stability

The stability of the standard solutions was investigated after storage for 7, 15, and 30 days at room temperature (25 °C) and under refrigeration (−5 °C) using the working solution.

#### 3.6.6. Robustness

The robustness of an analytical method is a measure of its capacity to resist changes due to small variations in parameter conditions, e.g., by using a different column. In this way, the method robustness was assessed as a function of changing the column brand for a C18 Agilent column (Agilent Technologies Inc, Santa Clara, CA, USA), (150 × 4.6 mm × 5 µm) and the ratio of the mobile phase.

### 3.7. Application of the Method

#### 3.7.1. Microemulsion Preparation

With the developed method in hand, the next step was to develop a stable microemulsion using a combination of clotrimazole and itraconazole. MEs were composed of benzyl alcohol, the non-ionic surfactant Tween^®^ 60, propylene glycol, and water. The optimum weight ratios of the components and MEs’ areas were determined using a pseudo-ternary phase diagram (data not shown in this work). The systems were prepared as previously described [22]; the surfactant (Tween^®^ 60) and cosolvent (propylene glycol) were prepared separately, and clotrimazole and itraconazol were solubilized in benzyl alcohol and added to the mixture. The pseudo-ternary phase diagrams of oil, surfactant/cosolvent, and water were set up using the water titration method.

#### 3.7.2. Stability Study

The stability profile of the prepared microemulsion at accelerated conditions was studied according to the ICH guidelines. The formulation was placed separately in an amber-colored screw-capped glass container and stored at 40 ± 2 °C and 5–8 ± 3 °C for 3 months, with sampling at 0, 30, 60, and 90 days. The samples were then evaluated for drug content using the developed HPLC method.

### 3.8. Characterization of the Synthetized Compounds

NMR spectra were obtained using a Varian Unity Plus VXR (Varian Inc., Walnut Creek, CA, USA), 500 MHz instrument in CDCl_3_ solutions. The chemical shifts were reported in units of d (ppm) downfield from tetramethylsilane, which was used as an internal standard; coupling constants (J) are reported in hertz and refer to apparent peak multiplicities. High-resolution mass spectra (HRMS) were recorded on a MICROMASS Q-TOF mass spectrometer (Waters, Milford, MA, USA).

## 4. Conclusions

The combination of clotrimazole and itraconazole in a pharmaceutical formulation is of great importance owing to the potential of generating a new option for the treatment of sporotrichosis. In this sense, the preformulation investigation using different techniques (DSC, TG, PXRD, FTIR) was essential to examine the existence of possible clotrimazole–itraconazole interactions.

Furthermore, an HPLC method was developed and validated according to standard guidelines, and it is the first reported method for the simultaneous determination of clotrimazole and itraconazole in nanotechnology-based products such as microemulsions. Based on our results, it was possible to conclude that there is no other co-eluting peak along with those of interest, the method being specific for the estimation of clotrimazole and itraconazole.

Interestingly, accelerated stability studies showed that a product derived from clotrimazole was formed, as well as a possible interaction between itraconazole and benzyl alcohol, when the microemulsion was conditioned at elevated temperatures (40 °C). On the other hand, the studies conducted at 5 °C showed that the microemulsion is stable for at least 3 months, as no degradation peaks were observed in the HPLC analysis, which allows us to infer that it is possible to guarantee the stability of the formulation under refrigeration.

## Figures and Tables

**Table 1:** Summary of the validation data obtained for the proposed HPLC method developed for the quantification of clotrimazole and itraconazole in microemulsions. LOD—limit of detection; LOQ—limit of quantification.

Standard Solutions	Parameters of the Method	Validation Results
Clotrimazole	Linearity	Calibration range (μg mL^−1^): 5–200 y = 233647.7939x − 312039.9299 (R^2^ = 0.9988)
	LOD	0.84 μg mL^−1^
	LOQ	2.54 μg mL^−1^
	Slope	233647.7939 ± 976.8015153
	Interception	−312039.9299 ± 59416.57811
Itraconazole	Linearity	Calibration range (μg mL^−1^): 5–160 y = 89946.6896x − 79996.5373 (R^2^ = 0.9999)
	LOD	0.86 μg mL^−1^
	LOQ	2.60 μg mL^−1^
	Slope	89946.6896 ± 780.1420761
	Interception	−79996.53731 ± 23351.48986

**Table 2:** Data related to the linearity of the developed HPLC method with its respective average, precision, and accuracy.

Concentration (µg/mL)	Clotrimazole			Itraconazole		
	Average (µg/mL)	Accuracy (%)	Precision (%)	Average (µg/mL)	Accuracy (%)	Precision (%)
5	4.883	97.7	0.20	5.593	111.9	0.86
10	9.292	92.9	0.57	10.115	101.2	0.91
20	19.233	96.2	0.40	20.029	100.1	0.58
40	38.927	97.3	0.01	39.621	99.1	1.12
80	77.731	97.2	1.08	79.154	98.9	1.81
160	151.888	94.9	0.71	160.488	100.3	0.82
200	204.631	102.3	0.65	-	-	-

**Table 3:** Data related to the repeatability and intermediate precision of the developed HPLC method.

Samples (μg mL^−1^)	Intra-Day Precision (Repeatability)			Inter-Day Precision (Intermediate Precision)		
**Clotrimazole**	**Concentration Found (μg mL^−1^)**	**Accuracy (%)**	**Precision (%)**	**Concentration Found (μg mL^−1^)**	**Accuracy (%)**	**Precision (%)**
7	6.818	97.4 ±2.25	0.47	6.865	98.07 ± 1.17	2.35
15	14.510	96.7 ±1.13	1.18	14.075	93.83 ± 3.17	0.95
120	116.679	97.2 ±0.27	0.28	121.108	100.92 ± 4.28	0.28
**Itraconazole**	**Concentration Found (μg mL^−1^)**	**Accuracy (%)**	**Precision (%)**	**Concentration Found (μg mL^−1^)**	**Accuracy (%)**	**Precision (%)**
7	7.206	102.9 ± 1.33	1.48	7.305	104.35 ± 1.25	1.20
70	70.809	101.2 ± 1.15	1.16	70.374	100.53 ± 2.41	2.40
150	152.745	101.8 ± 0.85	0.84	160.98	100.61 ± 4.9	1.59

**Table 4:** Data related to the stability of the assay of the developed HPLC method. N = 2 for each day and condition.

Days	Accuracy (%)	Precision (%)	Accuracy (%)	Precision (%)
Clotrimazole			Itraconazole	
**0**	97.3 ± 0.94 (25 °C)	1.18	101.4 ± 0.62	0.84
**7**	105.7 ± 0.89 (25 °C)	0.85	98.6 ± 4.48	4.57
	104.9 ± 0.07 (−5 °C)	0.07	98.4 ± 7.79	7.96
**15**	105.3 ± 1.51 (25 °C)	1.45	101.3 ± 0.51	0.50
	105.5 ± 0.39 (−5 °C)	0.38	100.1 ± 3.71	3.74
**30**	97.3 ± 3.15 (25 °C)	0.62	91.3 ± 2.71	3.21
	94.2 ± 0.34 (−5 °C)	0.73	88.7 ± 1.63	3.04

## Supplementary Materials

Supplementary materials are available online. Figure S1: Analytical calibration curve for clotrimazole, Figure S2: Analytical calibration curve for itraconazole, Figure S3: IR spectra of clotrimazole, itraconazole and their binary mixture (1:1), Figure S4: HPLC chromatogram of compound 3, Figure S5: HPLC chromatogram of the decomposition product formed via the forced degradation of clotrimazole in the presence of itraconazole.
