# Supplementary material for: Development of a Method for the Quantification of Clotrimazole and Itraconazole and Study of Their Stability in a New Microemulsion for the Treatment of Sporotrichosis
Source: Molecules. 2019 Jun 25;24(12):2333. doi: 10.3390/molecules24122333 (PMC6630458; doi:10.3390/molecules24122333)
Supplement: Supplementary file 1 [file molecules-24-02333-s001.pdf]

Supplementary data

Development of a method for the quantification of clotrimazole and itraconazole and study of their stability in a new microemulsion for the treatment of sporotrichosis

Patricia Garcia Ferreira[1], Carolina Guimarães de Souza Lima[2], Letícia Lorena Noronha[1], Marcela Cristina de Moraes[2], Fernando de Carvalho da Silva[2], Alessandra LifsitchViçosa[3], Débora Omena Futuro[1], Vitor Francisco Ferreira[1,*]

[1] Departamento de Tecnologia Farmacêutica, Faculdade de Farmácia, Universidade Federal Fluminense, Niterói-RJ 24241-000, Brazil; patricia.pharma@yahoo.com.br (P.G.F.); leticianoronha95@gmail.com (L.L.N.); dfuturo@id.uff.br (D.O.F.)

[2] Departamento de Química Orgânica, Instituto de Química, Universidade Federal Fluminense, Niterói-RJ 24210-141, Brazil; carolgslima@gmail.com (C.G.S.L.); mcmoraes@id.uff.br (M.C.M); gqofernando@vm.uff.br (F.C.S)

[3] Fundação Oswaldo Cruz (FIOCRUZ), Farmanguinhos – Manguinhos, Avenida Sinzenando Nabuco 100, Rio de Janeiro-RJ 21045-900, Brazil.

* Correspondence: vitorferreira@id.uff.br; Tel.: +55-21-998578148

(2-chlorophenyl)diphenylmethanol (3): Clotrimazole (50 mg, 0.14 mmol) was added to a round-bottom flask equipped with a stir bar. 10 mL of acetonitrile were then added to the flask, followed by the dropwise addition of concentrated hydrochloric acid (500 μL). The mixture was placed in an oil bath heated at 80 ˚C and stirred for 2h. Next, the solution was neutralized with a saturated sodium bicarbonate solution, the organic layer separated, and the aqueous phase extracted with ethyl acetate (3×). The combined organic layers were dried with anhydrous sodium sulfate and the solvent was removed in a rotary evaporator system. The product was purified using a silica gel column chromatography with a hexane:ethyl acetate (7:3) solution. White solid. 71% yield. ^{1}H-NMR (500MHz, $CDCl_3$): 1H-NMR (500 MHz, $CDCl_3$) δ 7.39 (dd, *J* = 7.9, 1.2 Hz, 1H), 7.36–7.21 (m, 11H), 7.10 (td, *J* = 7.7, 1.3 Hz, 1H), 6.70 (dd, *J* = 7.9, 1.6 Hz, 1H), 4.42 (s, 1H). HRMS Calculated: 294.08 Found: 277.0778 (M-OH).

(2-chlorophenyl)diphenylmethanol (3) – Formed via the forced degradation of clotrimazole in the presence of itraconazole. Clotrimazole (1 g, 2.9 mmol) and itraconazole (1 g, 1.4 mmol) were added to a mixture of benzyl alcohol (5 mL) and acetonitrile (5 mL) and stirred at 50 ˚C for 24 h. Next, the organic layer was separated, and the aqueous phase extracted with ethyl acetate (3×). The combined organic layers were dried with anhydrous sodium sulfate and the solvent was removed in a rotary

evaporator system. The product was purified using a silica gel column chromatography with a hexane:ethyl acetate (7:3) solution. White solid. 6% yield. HRMS Calculated: 294.08 Found: 277.0765 (M-OH).

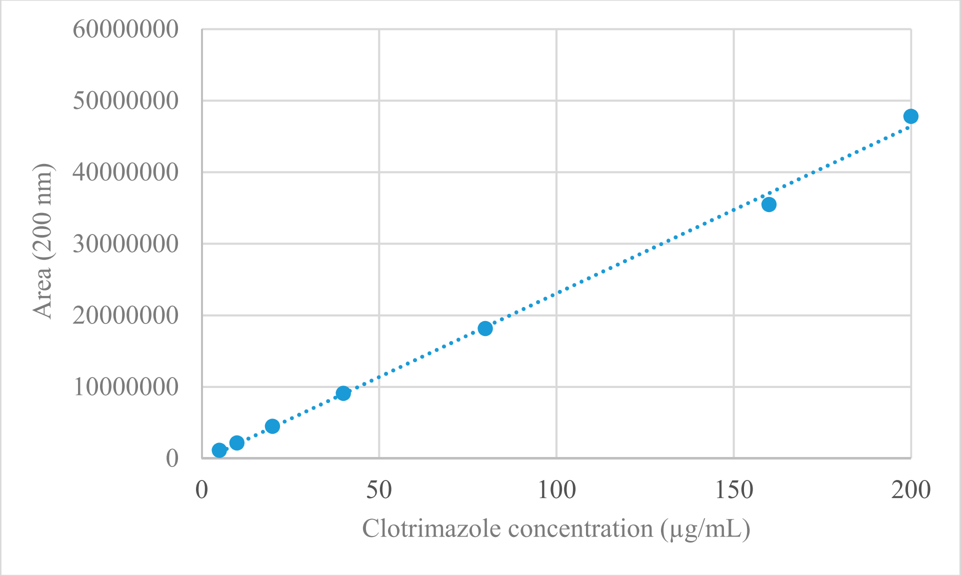

Figure S1. Analytical calibration curve for clotrimazole.

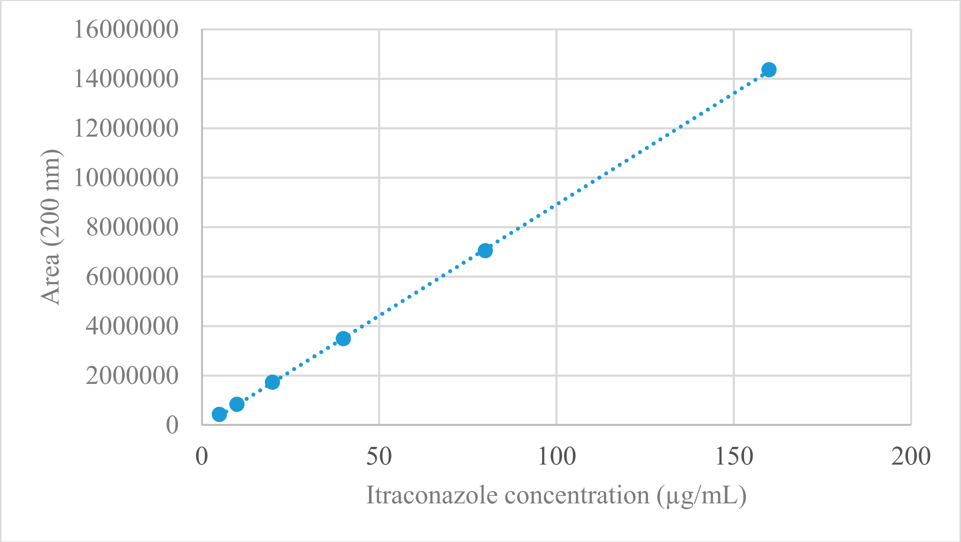

Figure S2. Analytical calibration curve for itraconazole.

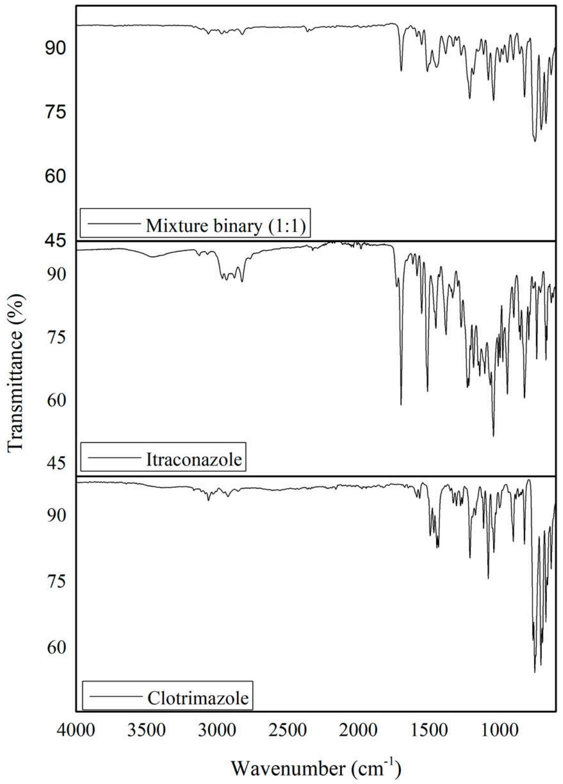

Figure S3. IR spectra of clotrimazole, itraconazole and their binary mixture (1:1).

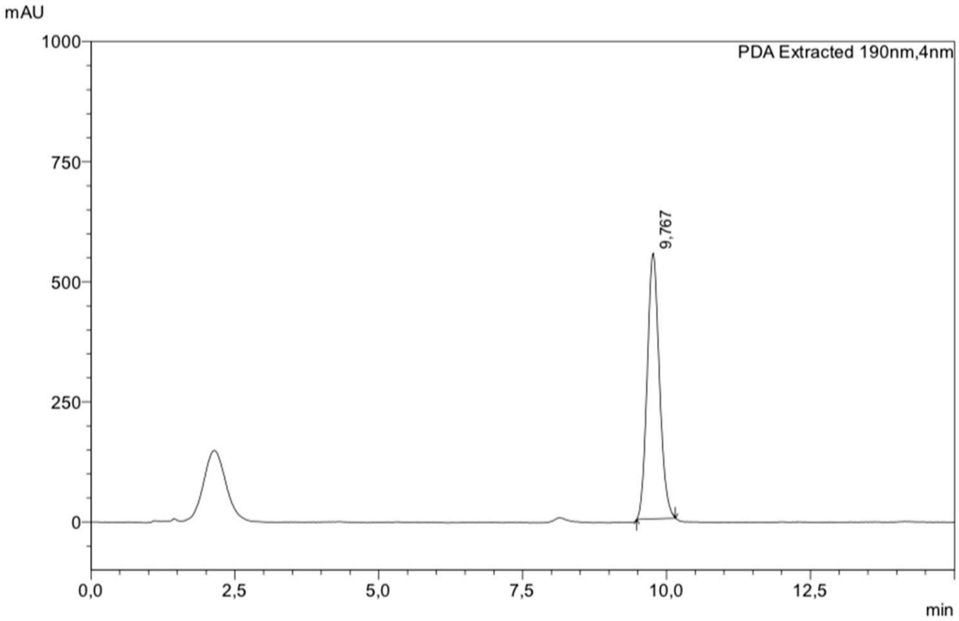

Figure S4. HPLC chromatogram of compound **3**.

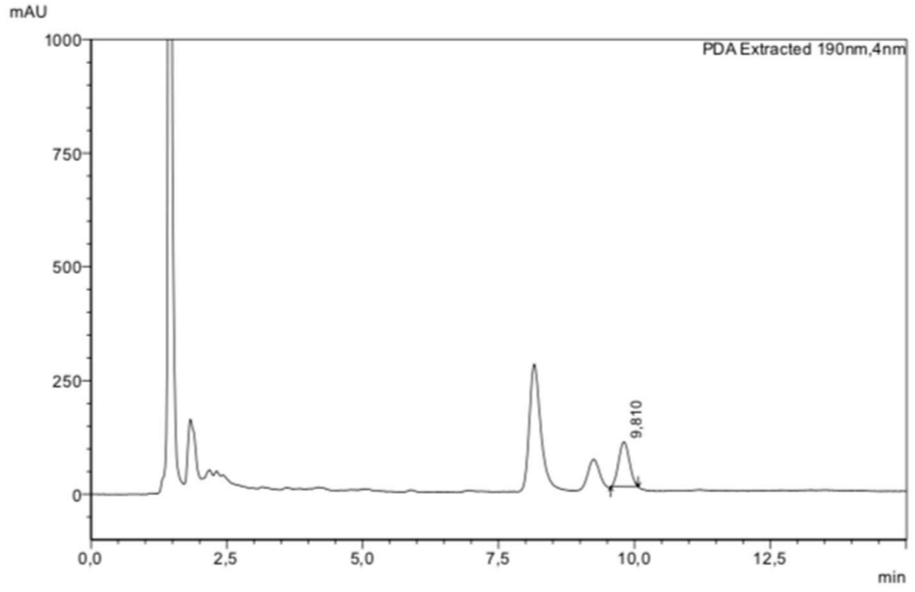

Figure S5. HPLC chromatogram of the decomposition product formed via the forced degradation of clotrimazole in the presence of itraconazole.
